# Inter-subject variability in the use of two different neuronal networks for reading aloud familiar words

**DOI:** 10.1016/j.neuroimage.2008.05.029

**Published:** 2008-09

**Authors:** M.L. Seghier, H.L. Lee, T. Schofield, C.L. Ellis, C.J. Price

**Affiliations:** Wellcome Trust Centre for Neuroimaging, Institute of Neurology, UCL, London, UK

**Keywords:** Functional MRI, Regional covariance, Inter-subject variability, Overt reading, Language, Connectivity, Network, Occipito-temporal sulcus

## Abstract

Cognitive models of reading predict that high frequency regular words can be read in more than one way. We investigated this hypothesis using functional MRI and covariance analysis in 43 healthy skilled readers. Our results dissociated two sets of regions that were differentially engaged across subjects who were reading the same familiar words. Some subjects showed more activation in left inferior frontal and anterior occipito-temporal regions while other subjects showed more activation in right inferior parietal and left posterior occipito-temporal regions. To explore the behavioural correlates of these systems, we measured the difference between reading speed for irregularly spelled words relative to pseudowords outside the scanner in fifteen of our subjects and correlated this measure with fMRI activation for reading familiar words. The faster the lexical reading the greater the activation in left posterior occipito-temporal and right inferior parietal regions. Conversely, the slower the lexical reading the greater the activation in left anterior occipito-temporal and left ventral inferior frontal regions. Thus, the double dissociation in irregular and pseudoword reading behaviour predicted the double dissociation in neuronal activation for reading familiar words. We discuss the implications of these results which may be important for understanding how reading is learnt in childhood or re-learnt following brain damage in adulthood.

## Introduction

Cognitive models of reading invariably include two or more possible mechanisms for translating written words into their spoken form. The segregation of these pathways is supported by neuropsychological observations of brain damaged patients who have striking dissociations in their ability to read different types of words (e.g. Coltheart et al., 1993, 1980; Newcombe and Marshall, 1973; Patterson and Hodges, 1992). For example, surface dyslexics have more difficulty reading “irregular words” with atypical spelling-to-sound relationships (e.g. CHOIR) than reading novel letter strings (e.g. CHOOP) that do not require lexical or semantic processing. In contrast, phonological dyslexics have the reverse dissociation. The observation that both types of dyslexics have relatively preserved reading of “regular” words with consistent spelling-to-sound relationships (e.g. “CHOP”) suggests that these words can be read either by direct translation of orthography to phonology (as in surface dyslexics) or via lexico-semantic associations of previously learnt words (as in phonological dyslexics). In short, cognitive models of reading suggest that there is more than one way to read regularly spelled words. Moreover, teaching methods or prior skills can bias an individual's reading strategy by generating a learning preference for either direct translation of letters to sounds or lexical and semantic associations (Connor et al., 2007; Rayner et al., 2001; Zevin and Balota, 2000). It is this inter-subject variability in reading familiar words that is the focus of the current study.

To investigate normal inter-subject variability in reading aloud familiar words, we used functional neuro-imaging. This allows us to look for a double dissociation in neuronal activation patterns for reading one type of word only. Based on the neuropsychological data and cognitive models discussed above, our hypothesis was that high frequency regular words could either be read using brain regions previously associated with reading novel pseudowords or brain regions previously associated with reading irregularly spelled words. To test this hypothesis, we aimed to (i) investigate inter-subject variability in reading activation that might reflect the use of different reading pathways across individuals, and (ii) categorize subjects in terms of their relative activation in areas previously associated with reading irregularly spelled words or pseudowords. We focus on the distinction between irregularly spelled words and pseudowords for two reasons: (i) it corresponds to the double dissociation observed in patients and (ii) it is less confounded by response time differences than the comparison of regularly spelled words and pseudowords (e.g. Fiebach et al., 2002; Fiez et al., 1999; Frost et al., 2005; Ischebeck et al., 2004; Joubert et al., 2004; Mechelli et al., 2003; Peng et al., 2004). Specifically our regions of interest are selected after consideration of the three studies that (i) use English words and involve skilled readers, (ii) use of a whole-brain analysis, and (iii) report a double dissociation in neuronal activation for reading irregularly spelled words and pseudowords (Binder et al., 2005; Herbster et al., 1997; Mechelli et al., 2005a).

The most consistent region associated with reading irregularly spelled words relative to pseudowords lies in the anterior occipito-temporal sulcus as shown in Table 1. The same region has also been associated with semantic reading (see Price and Mechelli (2005) for a review) and responded maximally to words relative to a range of nonwords (Vinckier et al., 2007). A second region consistently reported to be more activated for irregularly spelled words than pseudowords is the left ventral inferior frontal cortex. However, the coordinates of this effect have varied substantially over studies. For the reverse comparison (pseudowords relative to irregularly spelled words), the most consistently activated regions are the left dorsal premotor cortex and the left posterior occipito-temporal sulcus (see Table 1). The posterior occipito-temporal region is also the most consistent region associated with increased activation for pseudowords relative to regular word reading (see Mechelli et al. (2003) for a review).

In this context, we selected our regions of interest for pseudoword and irregular word reading in the left posterior and anterior occipito-temporal cortex respectively. The left occipito-temporal cortex has been the focus of attention in many other fMRI studies of reading (e.g. Baker et al., 2007; Reinke et al., 2008; Sandak et al., 2004; Vinckier et al., 2007). Previous work suggests that anterior and posterior parts of the left occipito-temporal cortex play different roles in word processing (Price and Mechelli, 2005; Vinckier et al., 2007) with the more posterior component thought to be involved at a perceptual level but the more anterior component involved at the semantic/lexical level (e.g. Dietz et al., 2005; Moore and Price, 1999; Peng et al., 2004; Price and Mechelli, 2005; Vigneau et al., 2006). In addition, connectivity analyses have suggested that the anterior occipito-temporal cortex is functionally connected to the ventral inferior frontal gyrus during irregular word reading whereas the posterior occipito-temporal cortex is functionally connected to the dorsal inferior frontal gyrus during nonword reading (Bokde et al., 2001; Mechelli et al., 2005a). Together, the evidence suggests that the anterior and the posterior parts of the left occipito-temporal cortex mediate different word processes.

To validate our choice of regions, we first investigated how activation in these regions covaried, across subjects, with that in the rest of the brain during regular word reading. As the task and word stimuli were held constant and each data point came from a different subject, covariance reflects inter-subject variability in reading activation (see Materials and methods section for more details). On the basis of the previous within-subject comparisons of irregular word and pseudoword reading discussed above, our prediction was that left anterior occipito-temporal (aOT) activation would covary with that in the left ventral inferior frontal cortex while left posterior occipito-temporal (pOT) activation would covary with that in the left dorsal premotor cortex. We then investigated whether inter-subject variability in the use of the aOT versus pOT networks during regular word reading corresponded to behavioural differences in how irregularly spelled words and pseudowords were read outside the MRI scanner. Our results show a between-subject double dissociation in activation for reading familiar words with some subjects showing more activation in areas associated with reading novel pseudowords and other subjects showing more activation in areas previously associated with reading irregular words.

## Materials and methods

The study was approved by the National Hospital for Neurology and Institute of Neurology Joint Ethics Committee.

### Subjects

Fifty-four healthy right-handed subjects (32 females, 22 males, aged 31.6 ± 20 years, range 13–74 years) gave written informed consent to participate in this study. Subjects were native English speakers with normal or corrected-to-normal vision, and no history of neurological or psychiatric disorders. Following data quality control checks (see below), 11 subjects (7 males and 4 females) were excluded. The final subject selection was therefore 43 (28 female, 15 male) in three distinct age groups: 13 adolescents (age range: 13–17); 18 young adults (age range: 20–34) and 12 older adults (age range: 48–74). We deliberately included several different age groups to maximize inter-subject variability. We were then able to explore whether the subject groups we dissociate could be explained by developmental differences in brain activation for reading (Booth et al., 2004; Chou et al., 2006; Shaywitz et al., 2007; Turkeltaub et al., 2003).

### Experimental design

During two separate scanning sessions, subjects read aloud a total of 96 three to six letter written object names. Although the present study focuses on the effect of reading aloud relative to fixation, two other conditions were also included: the first presented pictures of objects that subjects were instructed to name aloud (i.e. object naming); and the second involved visual presentation of meaningless pictures of non-objects or symbols in response to which subjects were instructed to say “1, 2, 3” (a visuo-motor baseline). In each of two scanning sessions/runs, there were four blocks of reading, four blocks of object naming, four blocks of the visuo-motor baseline, and six blocks of fixation baseline. Each lasted 18 s with 12 stimuli per block presented 3 at a time (i.e. in triads) for 4.5 s per triad. This enabled us to maximize presentation rate and paradigm efficiency. Items within the reading and object naming triads were selected such that there was no obvious semantic relationship between the three different items (e.g. slide, axe, cup). Condition order was fully counterbalanced within and across scanning session.

### Stimuli

All stimuli were derived from a set of 192 objects with three to six letter familiar names that had relatively consistent spelling-to-sound relationships: 33 had three letter names (cat, bus, hat), 65 had four letter names (ship, bell, frog, hand), 58 had five letter names (teeth, camel, snake) and 36 had six letter names (spider, dagger, button). A pilot study with 8 subjects ensured inter-subject agreement on all picture names. The 192 objects were first divided into two different sets of 96 items which we will refer to as Set A and Set B. One group of subjects (*N* = 22) was presented with set A as written words for reading aloud and set B as pictures for object naming. The other group (*N* = 21) was presented with set B as written words for reading aloud and set A as pictures for object naming. It was therefore necessary to test the influence of word set on any observed inter-subject variability in reading activation (see below for details). See Figure S2 in the supplementary materials for examples of triad stimuli.

Stimulus presentation was via a video projector, a front-projection screen and a system of mirrors fastened to a head coil. Words were presented in lower case Arial font, size 48 (maximum visual angle on retina = 4.9° × 1.2°). Pictures were all scaled to measure between 5–8 cm in width and height (maximum visual angle on retina = 7.3° × 8.5°). Accuracy of vocal responses during all conditions was recorded with a MRI-compatible microphone and a sound cancellation system. However, it was not possible to extract the response times. See below for procedures taken to minimize artifacts due to head motion during overt speech.

### MRI acquisition

Experiments were performed on a 1.5 T Siemens system (Siemens Medical Systems, Erlangen, Germany). Functional imaging consisted of an EPI GRE sequence (TR/TE/Flip = 3600 ms/50 ms/90°, FOV = 192 mm, matrix = 64 × 64, 40 axial slices, 2 mm thick with 1 mm gap). The EPI GRE sequence used here was optimized to minimize signal dropout by adjusting the slice tilt, the direction of the phase-encoding, and the *z*-shim moment (for more details see Weiskopf et al., 2006). Functional scanning was always preceded by 14.4 s of dummy scans to insure tissue steady-state magnetization. To avoid ghost-EPI artifacts, image reconstruction was based on a generalized algorithm (i.e. trajectory-based reconstruction after calibrating a trajectory scan during a gel-phantom experiment).

### First level analysis

Data processing and statistical analyses were carried out with Statistical Parametric Mapping SPM2 software package (Wellcome Trust Centre for Neuroimaging, London UK, http://www.fil.ion.ucl.ac.uk/spm/). All functional volumes were spatially realigned, unwarped, normalized to the MNI space, and smoothed with an isotropic 6 mm FWHM Gaussian kernel, with a resulting voxel size of 2 × 2 × 2 mm^3^. First level analyses of each subject's preprocessed data involved high-pass filtering (1/128 Hz cutoff) to remove low-frequency noise and signal drift from the time series in each voxel. Statistics were based on fixed-effect analysis using the general linear model in each voxel across the whole brain. Each stimulus onset was modelled as an event and convolved with a canonical hemodynamic response function (with no dispersion or temporal derivatives). For each subject, parameter estimates (i.e. beta images) were assessed with least square regression analysis, and the contrast images (i.e. weighted beta images) were computed for the main effect of reading aloud relative to fixation.

### Data quality control

It has been shown that overt reading may cause artifacts during fMRI data acquisition (e.g. Birn et al., 1998; Yetkin et al., 1996). We therefore performed a range of different control procedures to ensure the quality of the data. These precautions included short block durations (e.g. Soltysik and Hyde, 2006), optimized EPI sequence (Weiskopf et al., 2006), unwarping to correct artifacts caused by the interaction between head motion and geometric distortion, and asking subjects to whisper responses with minimal mouth movement. Under these methodological conditions, we were properly able to identify cortical regions involved in overt reading (e.g. Heim et al., 2006). Nevertheless, head motion was assessed for each subject by calculating the path length of the head motion for each block as previously proposed (D'Esposito et al., 1999) prior to normalization. Subjects were excluded if they had (i) a path length per block more than 1.5 mm, (ii) any parameter motion more than one voxel size (3 mm), or (iii) high signal loss artifacts. From this step, 11 subjects (7 males and 4 females) were excluded.

### Second-level covariance analysis

The covariance approach detailed below is comparable to that used in previous PET connectivity studies that searched the whole brain for regions that co-varied with activation in regions of interest (e.g. Horwitz et al., 1998; McIntosh, 1999). However, whereas these previous PET connectivity studies included both within and between subject variance, our second-level analyses were based on between subject variance only (one contrast image from each subject). In this sense, our approach is more comparable to the covariance analysis used with structural brain images (Mechelli et al., 2005b). The underlying rationale is that there is meaningful structure in the inter-subject variability (e.g. Kherif et al., 2003; Miller and Van Horn, 2007; Noppeney et al., 2006; Prat et al., 2007; Seghier et al., 2007) which can be explored by assuming that regions belonging to the same network will have comparable variations from subject to subject. In other words, regions that covary across subjects (i.e. their effect sizes going down and up across subjects) can be considered as part of the same network. This rationale has been recently used to identify cortical networks across subjects during rest (Damoiseaux et al., 2006), object recognition (Sugiura et al., 2007), words and symbol perception (Reinke et al., 2008), emotional memory suppression (Depue et al., 2007), and brain structure (Mechelli et al., 2005b). In short, we report the results of a standard method used in a novel context with new seed regions that were motivated by functional imaging studies of pseudoword and irregular word reading.

Our approach contrasts with other fMRI connectivity studies of reading that assess how activation in different regions correlate over time and how these correlations are modulated by experimental factors such as stimulus type (e.g. Bokde et al., 2001; He et al., 2003; Mechelli et al., 2005a, 2002; Prat et al., 2007; Zheng and Rajapakse, 2006). In other words, these studies investigated how the same subjects read different words, whereas our question concerned differences in the way that different subjects read the same words. For instance, functional connectivity at the group level during reading is usually assessed by averaging the data across subjects and then performing connectivity analyses (the notion of a “mean subject”, e.g. Bokde et al., (2001)), or by performing connectivity analyses within each subject (e.g. trial-by-trial or time-series analysis) and then combining the segregated networks or connectivity maps across subjects (e.g. Hampson et al., 2006; Mechelli et al., 2005a). Thus, while these analyses focused on within-subject variance in a limited set of regions, our analyses focused on between subject variance in each voxel across the whole brain. Covariance between regions in our approach therefore implies inter-subject variability. In addition, by searching the whole brain for areas that co-varied with aOT or pOT, our study has the potential to reveal brain areas that have not previously been investigated in prior functional connectivity studies of reading that were limited to a small set of regions.

### Step by step description of second-level data analyses

The contrast images for reading aloud relative to fixation from the first level analysis were analyzed at the second level as follows.

#### Analysis 1. Extracting each subject's activation in the aOT and pOT regions of interest

To extract the parameter estimates in anterior and posterior occipito-temporal regions (aOT and pOT respectively) during reading aloud, we entered the contrast images for reading relative to fixation into a one sample *t*-test. We then identified the peak coordinates closest to the regions of interest from Mechelli et al. (2005a) (on page 1756: anterior region: *x* = − 42 *y* = −42 *z* = − 18; posterior region: *x* = − 46 *y* = − 60 *z* = − 18). At our peak aOT and pOT coordinates, (*x* = − 44, *y* = − 44, *z* = − 16) and (*x* = − 44, *y* = − 68, *z* = − 18) respectively, we extracted the regional parameter estimates summarized as the principal eigenvariates of responses within a sphere (4 mm radius, 30 voxels). This resulted in two different vectors that were used in subsequent analyses. Critically, no outlier values were present in both regional parameter estimates according to the Hampel identifier (Hawkins, 1980). This is important because, like other univariate regression methods, our approach is inherently sensitive to the presence of outlier values (for a similar rationale, see Depue et al. (2007)).

#### Analysis 2. Dissociating reading networks that covary with aOT versus pOT activation

To identify brain regions that covaried with aOT or pOT activation during reading aloud, we used multiple regression. The contrast images were those for reading relative to fixation as in the previous analysis. In addition, the parameter estimates in aOT or pOT from the previous analysis were added as two separate covariates. This analysis allowed us to search the whole brain for regions where reading activation co-varied (increased and decreased) with that in either aOT or pOT.

Significant results are reported at *p* < 0.001 with correction for multiple comparisons (*p* < 0.05) made on the basis of extent (minimum cortical volume of 70 voxels per cluster).

#### Analysis 3. Influence of seed regions coordinates

To evaluate how the results from this analysis depended on the coordinates in the seed voxels of interest, we (i) divided the left occipito-temporal sulcus into 10 different sub-regions equally spaced along the anterior–posterior direction (MNI y from − 76 to − 40 mm) spanning our two regions of interest. The *x* and *z* coordinates were not manipulated (MNI *x* = − 44, MNI *z* = − 16) because they were relatively constant in our aOT and pOT region (*x* = − 44, *y* = − 44, *z* = − 16 and *x* = − 44, *y* = − 68, *z* = − 18 respectively); we then (ii) extracted the parameter estimates from Analysis 1 in each of the ten regions; (iii) regressed each of the ten parameter estimates with each of the regions identified in Analysis 2; (iv) plotted the parameter estimate for each regression to create the nine bar charts shown in Fig. 2.

#### Analysis 4. Classifying subjects on the basis of their reading behaviour outside the scanner

To investigate the behavioral correlates of aOT and pOT networks, we collected behavioral data in 15 adult subjects (9 females, 6 males, range 20–69 years). These subjects were selected because they were the last subjects to be scanned. After scanning, they were asked to read 50 irregular words (from the Wechsler Test of Adult Reading (Weschler, 2001)) and 20 pseudowords (from the Graded Nonword Reading Test (Snowling et al., 1996)) that were presented one at a time on a computer screen. Presentation rate was self paced with a button press response that prompted the next stimulus. Although this procedure meant that the resulting response times were longer than the speech production times, the measurement precision was held constant for both types of words tested. In other words, our analysis does not use these absolute response times (see below), it uses the difference between the same measure on two different types of words that were tested in the same way. In a typical behavioural experiment, we would counterbalance the order of conditions (e.g. irregular word and pseudoword reading). However, in this reading assessment, all subjects read the irregular words before the pseudowords. This was because (a) we did not know apriori whether a subject would be a fast or slow lexical reader; and (b) we did not want condition order to introduce inter-subject variability in the relative speed of irregular and pseudoword reading. Thus, a fixed order allowed us to keep the effect of the practice constant for all subjects and should therefore not be able to explain significant differences between subjects.

The relative difference in response time (RT) for irregular and pseudoword reading was then included as a regressor for a second-level analysis with SPM2. The contrast images of the 15 subjects were those for reading relative to fixation as in Analysis 1. Our regions of interest were those that showed a significant difference in covariance between aOT and pOT in Analysis 2 (see right hand column of Table 2). Significant effects were identified by (i) limiting the search space to 10 mm from the peak coordinates in Table 2; and (ii) reporting peaks with *Z* ≥ 3.0 with the number of voxels at *p* < 0.01.

## Results

All imaging results were obtained from 43 healthy right-handed subjects (28 females, 15 males). Subjects were instructed to read aloud familiar words with three to six letters and consistent spelling-to-sound relationships (e.g. bus, basket). Task accuracy was 99 ± 1%.

### Analysis 1. Extracting each subject's activation in the aOT and pOT regions of interest

**Fig. 1:**
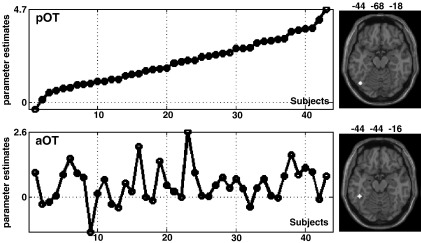
Left: Parameter estimates in the left pOT and aOT seed voxels. For illustration purposes, subjects were sorted according to their activation in pOT. The horizontal dashed line represents zero activation. Right: The locations of the pOT and aOT voxels (used as seed regions) are drawn on an axial slice from a canonical brain. A scatter plot of pOT versus aOT activation is shown in Figure S1 of the supplementary material.

Each subject's contrast image for reading aloud high frequency regularly spelled words relative to fixation was entered into a one sample *t*-test (i.e. activation was pooled irrespective of subject age or gender). This analysis confirmed that activation for reading aloud relative to fixation was observed in distributed occipital, temporal and frontal regions as previously described (e.g. Binder et al., 2005; Sandak et al., 2004; Turkeltaub et al., 2002). In the left occipito-temporal sulcus, posterior (pOT) and anterior (aOT) occipito-temporal regions of interest were localized at coordinates (*x* = − 44 *y* = − 68 *z* = − 18; *Z* score = 7.5) and (*x* = − 44 *y* = − 44 *z* = − 16, *Z* score = 3.3) respectively. The parameter estimates for each subject at each of these coordinates were extracted from this first analysis as a measure of pOT and aOT reading response. These parameter estimates were then used as regressors in the second analysis. The independence of these regressors is illustrated in Fig. 1 which shows that effect sizes varied with both the subject and the seed voxel. In fact, there was no significant correlation between activation in aOT and pOT (*r* = 0.15, *p* > 0.1). This suggests that, despite being part of the same anatomical gyrus, aOT and pOT responses may be independent of one another across subjects, consistent with these regions participating in different reading processes.

### Analysis 2. Dissociating reading networks that covary with aOT versus pOT activation

**Fig. 2:**
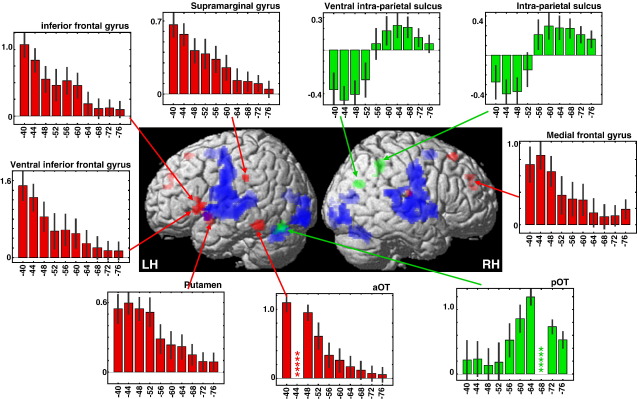
3D rendering of left (LH) and right (RH) hemisphere reading activation showing regions that covaried with aOT more than pOT (red), pOT more than aOT (green) and regions from the main effect of reading aloud relative to fixation that did not show significant covariance with either aOT or pOT (blue). The bar graphs show the effect size (± SE) in each region of the aOT and pOT networks when the seed voxel is moved from anterior (MNI *y* = − 40 mm) to posterior (MNI *y* = − 76 mm) OT. The localization (MNI *xyz* coordinates) of each region is indicated in the top-right of each bar graph. For illustration purposes, regions with a size less than 20 voxels from the comparison “aOT > pOT” or “pOT > aOT” are not shown.

The second analysis extended on the first by including the aOT and pOT parameter estimates as regressors of interest (i.e. multiple regression analysis). This enabled us to identify brain regions where reading activation covaried with that in aOT more than pOT or vice versa (see Table 2 and Fig. 2). The regions where reading activation covaried with that in aOT more than pOT included left ventral inferior frontal cortex, medial frontal cortex, left supramarginal cortex and the left putamen. From here on, we refer to these regions as the aOT network. In contrast, activation in bilateral intraparietal cortex was significantly more correlated with pOT than aOT. We refer to these regions as the pOT network. In the left dorsal premotor area associated with pseudoword reading, activation covaried with that in pOT (as expected) but this effect was not significantly greater for pOT than aOT. The aOT and pOT networks are illustrated in Fig. 2 in red and green respectively. In addition, the blue areas in Fig. 2 are those that were significant for the main effect of reading relative to fixation but did not show significant covariance with either aOT or pOT. They include bilateral visual, motor and auditory areas that support reading aloud in all subjects (see Table S2 in the supplementary materials for a full list of coordinates).

To ensure that the segregation of aOT and pOT networks is not related to other variables, we examined the correlations (with simple regression analyses) of age, gender and word set on activation in the aOT and pOT seed regions (see Table 3). Of these variables, only age had a significant (*p* < 0.05) effect on aOT, such that activation was higher in the aOT network for younger subjects. This is in line with previous work that showed stronger involvement of OT in young subjects (e.g. Balsamo et al., 2006). Critically, however, the effect of age in the aOT network can not account for the double dissociation in the aOT and pOT networks reported in Table 2. If it had, then there should be a positive correlation of age in pOT but this was not observed. To the contrary the correlation of age in pOT was non-significantly negative rather than positive. Likewise, although there was a trend for higher aOT activation in males than females (*p* < 0.08), this can not explain the segregation of the aOT and pOT networks.

### Analysis 3. Influence of seed region location on aOT and pOT networks

The bar graphs in Fig. 2 illustrate how covariance in each region varies with different subdivisions of the left OT. In the aOT network, covariance decreases in a step wise function as the seed voxel moves from aOT to pOT. By contrast, in the pOT network, covariance decreases in a step wise function as the seed voxel moves from pOT to aOT. These observations are important because they (i) indicate that the segregated neuronal systems would still be apparent if our seed voxels were shifted a few voxels either way along the occipito-temporal sulcus, and (ii) illustrate the specificity of the aOT and pOT subdivisions.

### Analysis 4. Classifying subjects on the basis of their reading behaviour outside the scanner

**Fig. 3:**
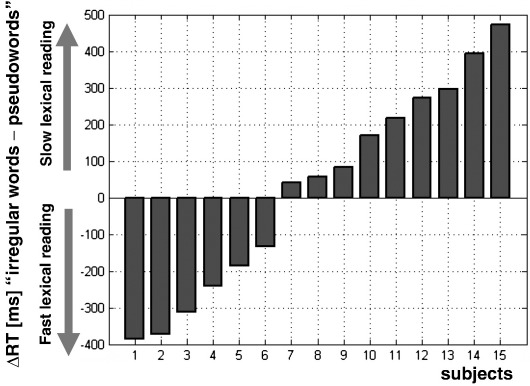
Differences in RTs [ms] between reading irregular words versus pseudowords in each of the 15 subjects with behavioural data. For illustration purposes, subjects were sorted from fast lexical readers (ΔRT < 0, e.g. subject 1) to slow lexical readers (ΔRT > 0, e.g. subject 15).

In this section, we classify subjects on the basis of their reading behaviour outside the scanner. Our aim is to use individual differences, within skilled readers, as a way of revealing different mechanisms that might be involved in reading aloud written words. For a similar rationale, see Baron and Strawson (1976) and Freebody and Byrne (1988). Fifteen of the 43 subjects read 50 words with irregular spellings (e.g. ‘aisle’, ‘ballet’, ‘ogre’) and 20 pseudowords (e.g. ‘gromp’ ‘tegwop’, ‘kipthirm’). Subject selection was unbiased in so much as they were the last to be scanned. The accuracy and mean RTs for reading irregular words and pseudowords were not significantly different (see Table S3 of the supplementary material). However, subjects showed notable differences in response time during reading irregular words versus pseudowords: six subjects read irregular words faster (on average) than pseudowords whereas nine subjects had the opposite pattern. These behavioural differences can not be explained by order effects because all the subjects had the same order (i.e. all subjects read the irregular words before the pseudowords), see methods for the rationale of this atypical procedure. Fig. 3 illustrates the differences in response times for the 15 subjects.

Using second-level regression analysis, we then investigated whether reading activation in the 15 subjects with behavioural data correlated with the difference in their response time to read irregular words and pseudowords. We found that slow lexical reading (i.e. positive difference in RTs for irregular relative to pseudoword reading) showed more activation in the aOT network while fast lexical reading (i.e. negative difference in RTs for irregular relative to pseudoword reading) showed more activation in the pOT network (see Table 4). We are therefore able to link differential activation of reading systems (i.e. pOT and aOT) to a double dissociation in reading behavior (i.e. fast versus slow lexical reading).

## Discussion

In this study, we hypothesized that reading high frequency words with relatively consistent spelling-to-sound relationships could either be supported by brain regions previously associated with reading irregularly spelled words or brain regions previously associated with reading unknown pseudowords. To this end, we first explored the network of brain regions that co-varied across subjects with two “seed” regions previously associated with irregularly spelled words versus pseudowords. This demonstrated a double dissociation in what we refer to as the aOT and pOT networks. This dissociation could not be explained by gender, age or stimulus differences but it could be explained by a double dissociation in reading speed for irregularly spelled words versus pseudowords. Below we discuss the dissociated networks in relation to both anatomical and cognitive models of reading.

### The aOT and pOT networks

The covariance analysis demonstrated that subjects with relatively high reading activation in aOT but not pOT also showed relatively high reading activation in the left ventral inferior frontal cortex, left putamen, left supramarginal gyrus and medial superior frontal cortex. Of this set, only the left ventral inferior frontal cortex was predicted on the basis of previous studies comparing irregularly spelled words to pseudowords. The coordinates we observed in this region (Table 2) were close to those reported for irregular word reading by Herbster et al. (1997) and Binder et al. (2005) (see Table 1 above). They are also close to those associated with semantic processing of written words in numerous other studies (e.g. Roskies et al., 2001; Seghier et al., 2004). In addition, Devlin et al. (2003) reported that TMS directed at (*x* = − 52, *y* = 24, *z* = − 2) significantly delayed semantic decision times, and Vigneau et al. (2006) identified a centre of mass at (*x* = − 43, *y* = 21, *z* = 4) in a meta-analysis of previous semantic studies. Likewise, the medial frontal region (Table 2) where we found activation covaried with that in aOT has also been associated with semantic processing (Chan et al., 2004; McDermott et al., 2003; Mummery et al., 1998, 1999; Poldrack et al., 1999; Sakurai et al., 1992; Thuy et al., 2004; Usui et al., 2003). For example, direct comparison of semantic and phonological tasks resulted in medial frontal activation at (*x* = − 5, *y* = 55, *z* = 20) in Poldrack et al. (1999) and (*x* = − 13, *y* = 47, *z* = 30) in Roskies et al. (2001). Together co-activation in aOT, left ventral inferior frontal and medial frontal regions suggests increased demands on semantic processing.

Contrary to our expectations, however, the pOT network did not include areas associated with phonological processing. For example, the left ventral supramarginal gyrus and left putamen are typically associated with phonological and speech output processes (e.g. Riecker et al., 2005; Wise et al., 1999) yet these regions covaried more strongly with aOT than pOT. Indeed, the left supramarginal region associated with the aOT network corresponded almost exactly to that reported by Mummery et al. (1998) (at *x* = − 56, *y* = − 34, *z* = 34) and Seghier et al. (2004) (at *x* = − 56, *y* = − 38, *z* = 36) for phonological compared to semantic decisions. For the pOT network, in contrast, we identified bilateral regions in the intraparietal sulci that have previously been associated with visual processing and attention (Corbetta et al., 1993; Dong et al., 2000; Gitelman et al., 1999; Pammer et al., 2006; Ravizza et al., 2005). This is comparable to the findings of Gitelman et al. (1999) who identified an attentional network connecting bilateral parietal cortex to the occipito-temporal cortex. Although these intraparietal areas are not associated with phonological processing, it is noteworthy that Binder et al. (2005) observed increased activation in these regions (at *x* = − 22, *y* = − 69, *z* = 42; *x* = − 33, *y* = − 51, *z* = 39; *x* = 22, *y* = − 66, *z* = 46; *x* = 25, *y* = − 67, *z* = 31) for pseudoword reading compared to irregular word reading, and Valdois et al. (2006) observed increased activation at (*x* = − 24, *y* = − 59, *z* = 54; *x* = 32, *y* = − 56, *z* = 53) during reading long (i.e. polysyllabic) pseudowords (Baciu et al., 2002; Valdois et al., 2006). Thus the double dissociation in left ventral inferior frontal and bilateral intraparietal activation that we observed – between subjects – for regular word reading has previously been reported by Binder et al. (2005) – within subjects – for reading aloud irregular words and pseudowords respectively.

With respect to the independence of the aOT and pOT system, Fig. 2 illustrates that activation in the intraparietal regions increases with pOT activation but decreases with aOT activation. This negative correlation of intraparietal activation with the aOT system suggests two independent systems that might compensate for one another. Thus, a patient with damage to one system might attempt to compensate with increased use of the other system. However, Fig. 2 also illustrates that aOT and pOT activation were neither negatively nor positively correlated. Moreover, Fig. 1 illustrates that all but one of our 43 subjects showed above zero reading activation in pOT but only 65% of our subjects showed above zero reading activation in aOT. It may therefore be the case that pOT activation feeds both the aOT and the pOT networks.

To explore the relationship between the two networks further, we divided subjects into four different groups according to their relative aOT and pOT activation (see Figure S1 of the supplementary material). This demonstrated that some subjects show high activation in both pOT and aOT, some show low activation in both regions and some show high activation in one region and low activation in another region. Thus, the use of one reading network does not preclude the use of another and, consistent with cognitive models of reading (e.g. Seidenberg and McClelland, 1989), both systems could theoretically be activated in parallel.

In summary, our results suggest that components of the aOT and pOT networks are functionally dissociable from one another but this does not mean the networks are mutually exclusive of one another. Nor does it mean that either of these systems can function independently from the shared set of regions that did not correlate with either aOT or pOT. These shared regions include sensori-motor areas (e.g. bilateral occipital cortex, motor and premotor regions) that are likely to be necessary for all reading networks. In other words, during regular word reading, some regions were activated by all subjects, whereas others (e.g. aOT or the intraparietal areas) responded independently across subjects in a continuous fashion. We also note that the dependence and independence of these networks is likely to change with both the stimuli and the task (e.g. Zevin and Balota, 2000) but future studies are required to investigate this issue.

### Inter-subject variability in reading behaviour

In the discussion above we attempted to define the functions of the different networks by reference to the results of previous functional imaging studies. We argued that the double dissociation in reading activation for the aOT and pOT networks is consistent with a double dissociation at the cognitive level between semantic and phonological processing (the aOT network) and a visual-attention system (the pOT network) even though we did not manipulate either semantic demand or visual attention. There are of course limits to using “reverse inference” to identify the functions of different brain regions (Poldrack, 2006). We therefore also explored the cognitive correlates of aOT and pOT systems on the basis of irregular and pseudoword reading behaviour, outside the scanner in a subset of our subjects.

Our behavioural measure of interest was each subject's relative speed reading irregularly spelled words and pseudowords. Some subjects read irregular words faster than pseudowords (i.e. fast lexical readers) whereas other subjects read irregular words slower than pseudowords (i.e. slow lexical readers). We then correlated regular word reading activation with these differences in responses times. Slow lexical reading showed more activation in regions of the aOT network associated with semantic processing (aOT, left ventral inferior frontal cortex and medial frontal cortex), see Table 4. In contrast, fast lexical reading showed more activation in the pOT network associated with visual attention. This suggests that the semantic system is more activated by slow lexical readers and the visuo-attention system is more activated by fast lexical readers. Initially we were surprised by this result because we expected fast lexical reading to increase reliance on the semantic (aOT) network and slow lexical reading to increase reliance on the visuo-attention (pOT) network. There is, however, an alternative interpretation which is that increased activation may reflect more effort, not more efficiency (e.g. Prat et al., 2007). Indeed, other studies have observed that activation in semantic regions is greater for words with weaker semantic associations, consistent with more difficulty accessing semantic representations (Chou et al., 2006), (though see Drager et al. (2004) and Rypma et al. (2005)). Likewise, semantic activation can be stronger for pseudoword reading than regular word reading because subjects are delayed searching for an unavailable semantic solution (see Forster and Bednall, 1976; Price et al., 1996). This may explain why some studies have observed increased activation in the left ventral inferior frontal cortex for pseudowords more than regular words (e.g. Fiebach et al., 2002; Fiez et al., 1999; Hagoort and Indefrey, 1999; Heim et al., 2005; Joubert et al., 2004). Our point here is that slow lexical readers may use the aOT semantic network inefficiently whereas fast lexical readers may use the pOT attentional network inefficiently. Top-down modulation may also increase for inefficient word processing (e.g. de Zubicaray et al., 2006; Noesselt et al., 2003). These behavioural results therefore highlight the fact that the aOT and POT networks are not mutually exclusive. Both networks appear to work in parallel to support different aspects of reading and subjects differ in their relative efficiency of the two networks.

### Other reading pathways

With respect to other neuronal models of reading, Pugh et al. (2000) have suggested a left hemisphere ventral processing stream that specializes in lexical activation for familiar words and a left hemisphere dorsal stream that is specialized for sub-word analysis (i.e. mapping spelling-to-sound for less familiar letter strings). As reading skill develops, the ventral regions are predicted to become increasingly activated. This is consistent with dual-route cognitive models of reading (e.g. Coltheart et al., 1993) where a lexical pathway directly maps orthographic percepts to stored word form representations and a sublexical pathway translates graphemic input to phonological output. Both pathways are activated in parallel but the lexical pathway is faster for high frequency words while the sublexical pathway is more involved for low-frequency words (e.g. Paap and Noel, 1991; Visser and Besner, 2001). Our experiment was not designed to test the model presented in Pugh et al. (2000). All our subjects were skilled readers and we were looking for dissociations within the ventral processing stream. We therefore treated aOT and pOT as parts of different networks whereas in the Pugh et al. (2000) model, these regions are treated as one (see for example Sandak et al. (2004)). Moreover, we found a double dissociation between the posterior parietal regions associated with the pOT network and the more anterior left parietal region (in the supramarginal gyrus) associated with the aOT network. In short, Pugh et al. (2000) focus on the difference between skilled and unskilled reading whereas we are reporting differences within a skilled reading group. Potentially, the differences at the neuronal level that we found here might mirror the previously reported differences at the behavioural level within skilled readers (e.g. Baron and Strawson, 1976; Hyona and Nurminen, 2006; Zevin and Balota, 2000).

### Implications and further studies

Several new questions are generated by our results. For example, what factors determine which reading system an individual will activate? The answer to this question might relate to genetic factors, contrasting methods in learning, phonological abilities or prior reading experience (e.g. Connor et al., 2007; Kouri et al., 2006; Prat et al., 2007; Pugh et al., 2001; Rayner et al., 2001; Sandak et al., 2004; Shaywitz et al., 2004). We are also extending the current study of regular word reading to investigate covariance for reading irregular words and pseudowords. In addition, we are exploring different seed regions to investigate other dissociable reading networks, for example, the insula and the inferior frontal cortex (e.g. Bokde et al., 2001; Borowsky et al., 2006; Raichle et al., 1994). On the anatomical level, our DTI tractography studies are currently exploring the white matter connections to and from pOT and aOT and our patient studies are comparing the effect of damage to either the aOT system, the pOT system or both.

Irrespective of the answers to these questions, several new conclusions can be validly made from our results at this stage. First, we have dissociated two different neuronal networks that activate when familiar regularly spelled words are read. Second, the inter-subject variability in neuronal systems corresponds to a behavioural dissociation in how irregular words and pseudowords are read. Most importantly, however, our results generate very precise predictions about how reading will survive or recover in patients who have suffered neurological damage to one or more of the identified regions. Specifically, we predict that recovery from damage to a component of one reading system will depend on the integrity of the surviving system. Thus damage to both systems is likely to be more disruptive than damage to several parts of one system.

## Figures and Tables

**Table 1:** List of coordinates for the most consistently activated regions in three previous studies that compared irregular word to pseudoword reading

Contrast of interest	Region	Coordinates (*x y z*)			Study
Irregular word > pseudoword	anterior occipito-temporal sulcus	− 38	− 40	− 24	Herbster et al., 1997
− 42	− 42	− 18	Mechelli (2005a)
inferior frontal cortex	− 40	12	− 4	Herbster et al., 1997
− 52	32	4	Mechelli (2005a)
− 39	25	− 9	Binder (2005)
Pseudoword > irregular word	posterior occipito-temporal sulcus	− 46	− 60	− 18	Mechelli (2005a)
− 49	− 63	− 11	Binder (2005)
dorsal premotor cortex	− 56	0	40	Mechelli (2005a)
− 48	− 12	44	Binder (2005)

**Table 2:** List of regions where activation covaries with that in either the anterior (aOT) or posterior (pOT) occipito-temporal regions of interest (at *p* < 0.05 corrected for multiple comparisons across the whole brain in either height or number of voxels at *p* < 0.001 uncorrected)

Regions that covary with	MNI coordinates			*Z* scores and cluster size	
				Main effect	Relative effect
*aOT*				*aOT*	*aOT > pOT*
Left anterior occipito-temporal sulcus	− 44	− 44	− 16	Inf; 256 ⁎⁎	Inf ⁎⁎
Left ventral inferior frontal gyrus	− 50	16	8	4.1; 160 ⁎⁎	3.6; 91 ⁎⁎
	− 50	18	− 4	3.9	3.8
Medial frontal gyrus	0	42	34	4.7; 230 ⁎⁎	3.8
	0	50	24	4.3	3.9; 56
Left supramarginal gyrus	− 58	− 34	34	4.4; 74 ⁎⁎	3.9; 23
Left putamen	− 26	10	− 4	4.9; 238 ⁎⁎	4.8; 61

*pOT*				*pOT*	*pOT > aOT*
Left posterior occipito-temporal sulcus	− 44	− 68	− 18	Inf; 383 ⁎⁎	7.8 ⁎⁎
Left dorsal premotor cortex	− 42	6	46	4.4; 61	(ns)
Right intraparietal sulcus	40	− 72	30	4.7; 489 ⁎⁎	3.5
	30	− 68	26	3.9	4.7; 58
	30	− 60	36	4.7	3.1
	28	− 50	46	3.9	4.3; 102 ⁎⁎
	34	− 44	48	4.5	3.8
Left intraparietal sulcus	− 30	− 72	20	4.6; 523 ⁎⁎	3.4; 7
	− 22	− 62	46	4.4	(ns)
	− 34	− 60	42	4.2	3.3
	− 26	− 58	36	3.6	3.4; 11

(ns): not significant at *p* < 0.001 uncorrected. ⁎⁎: significant clusters at the corrected level of *p* < 0.05.

**Table 3:** Correlation between aOT and pOT activation with age, gender and word set

	Age	Gender	Word set
aOT	*r* = − 0.31; *p* = **0.04**	*r* = − 0.27; *p* = 0.08	*r* = − 0.15; *p* = 0.33
pOT	*r* = − 0.15; *p* = 0.33	*r* = − 0.02; *p* = 0.88	*r* = − 0.11; *p* = 0.49
aOT – pOT	*r* = − 0.04; *p* = 0.81	*r* = − 0.13; *p* = 0.40	*r* = 0.01; *p* = 0.93

The *p* values (d*f* = 41) indicate the significance of these correlations being different from zero (bold = significant correlation at *p*<0.05).

**Table 4:** Results of the analysis regressing activation for reading aloud relative to fixation with the difference in response times for irregular and pseudoword reading (subset of 15 subjects only)

Regions	MNI coordinates			*Z* score; size
*Positive correlation: slow lexical reading*				
Left anterior occipito-temporal sulcus	− 46	− 38	− 14	3.4; 14
Left ventral inferior frontal gyrus	− 46	6	4	3.2; 23
	− 46	10	2	3.1
Left putamen	− 26	2	− 10	3.0; 26
Medial frontal gyrus	2	46	26	3.7; 121

*Negative correlation: fast lexical reading*				
Left posterior occipito-temporal sulcus	− 40	− 74	− 10	3.0; 18
Right intraparietal sulcus	28	− 64	34	3.2; 17
	38	− 72	22	3.1; 25
Left intraparietal sulcus	− 32	− 60	40	3.6; 62
	− 28	− 80	18	3.4; 40

Effects are reported within the regions of interest listed in Table 2.
