# Supplementary material for: Inter-subject variability in the use of two different neuronal networks for reading aloud familiar words
Source: Neuroimage. 2008 Sep;42(3-3):1226–36. doi: 10.1016/j.neuroimage.2008.05.029 (PMC2724104; doi:10.1016/j.neuroimage.2008.05.029)
Supplement: Supplementary file 1 [file mmc1.pdf]

Post hoc analysis of the covariance results

The inter-subject variability implied from the covariance analysis (Analysis 2) could be expressed in different ways. For example, if subjects used either the aOT or pOT networks (i.e one was mutually exclusive of the other), then we would expect a negative relationship between aOT and pOT activation across subjects. This was not the case. Instead we found that some subjects activate one network more than the other while others show strong or weak activation in both networks. We therefore categorized our subjects according to their relative activation in aOT and pOT. This allowed us to demonstrate a categorical difference in the degree to which different subject groups activated the aOT and pOT networks respectively.

Subjects were classified into four different groups according to whether their aOT or pOT activation was high (above the mean for all subjects) or low (below the mean for all subjects). The four different subgroups were: (i) Group HH who had high (H) activation in both regions (11 subjects); (ii) Group LL who had low (L) activation in both regions (12 subjects); (iii) Group HL who had high activation in aOT but low activation in pOT (9 subjects); and (iv) Group LH who had low activation in aOT but high activation in pOT (11 subjects). See Figure S1 for more details.

It was the difference between Groups HL and LH that drove the dissociation between aOT and pOT networks. Indeed, a direct comparison of the 9 subjects in Group HL and the 11 subjects in Group LH (using a two sample t-test with no regressors) confirms that Group HL activated the aOT network more than Group LH while Group LH activated the pOT network more than Group HL (see Table S1). Note that this 2 sample t-test with small subject groups is less sensitive than the covariance analysis because (i) subjects varied along a continuum of aOT versus pOT activation (rather than there being a categorical distinction between subject groups), (ii) the covariance analysis identified significant regions in aOT or pOT networks irrespective of the size of their mean main effects (whereas the results of the two sample t-test are dependent on the size of mean differences), and (iii) there were fewer degrees of freedom with the reduction of subjects (20 subjects from the original pool of 43 subjects). Nevertheless, the results of the 2-sample t-test (at $p<0.01$ uncorrected) support the across-subject correlation findings.

Figure S1: scatter plot showing the parameter estimates of aOT versus the parameter estimates of pOT. The four quadrants (HH, LL, Hl and LH) are illustrated. Each subject is represented by one black dot. The horizontal and vertical dashed lines represent the mean over subjects of the parameter estimates of aOT and pOT respectively. Correlation between aOt and pOT is indicated in the top right corner.

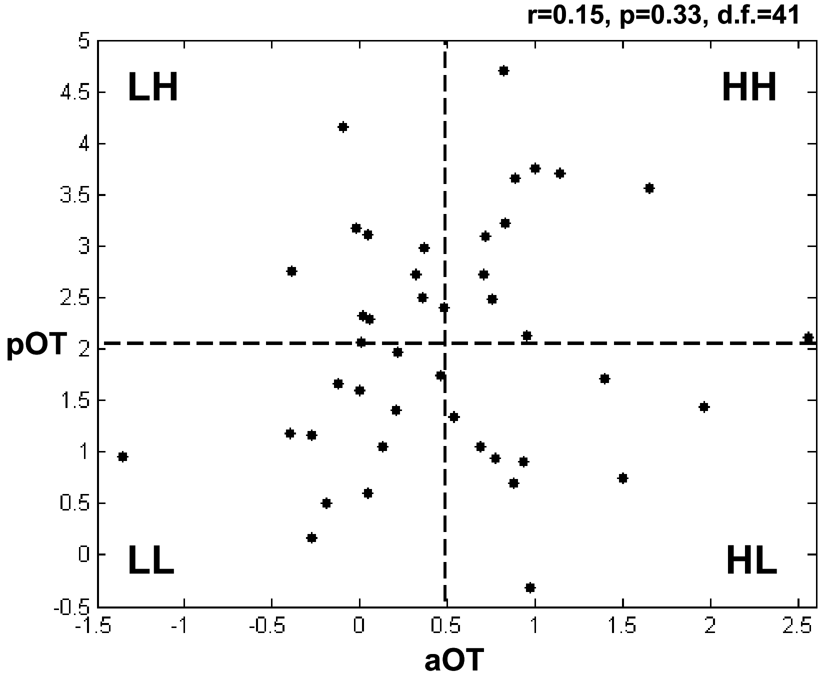

Table S1: Comparison between HL (high aOT activation but low pOT activation) and LH (low aOT activation but high pOT activation) groups. Effects of interest were reported within the regions listed in Table 2 of the manuscript. Only regions with Z-score ≥ 3.0 are reported.

	MNI coordinates	Z-score
**<u>Group HL > Group LH</u>**		
Left anterior occipito-temporal sulcus	-42 -44 -16	4.7
Left ventral inferior frontal gyrus	-56 16 2	3.1
Left putamen	-28 2 -12	3.3
**<u>Group LH > Group HL</u>**		
Left posterior occipito-temporal sulcus	-42 -68 -18	4.9
Right intra parietal sulcus	38 -72 28	3.2
	32 -66 24	4.2
	26 -50 40	4.1
Left intraparietal sulcus	-32 -72 18	3.7
	-34 -60 40	3.2

Figure S2: Examples of four word triad stimuli. Subjects were instructed to read the top word first followed by the lower left and then the lower right, as they would if they were reading text.

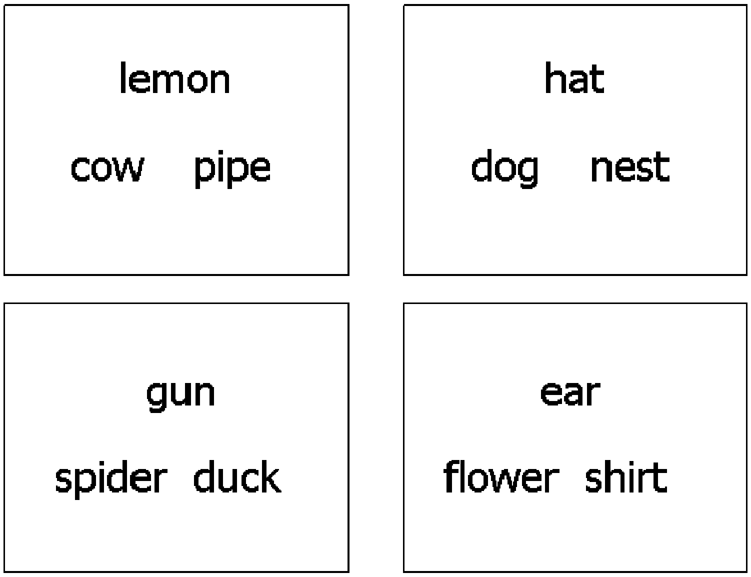

Table S2: List of all significant local maxima of the main effect of reading aloud relative to fixation (second level analysis on 43 subjects, p<0.05 corrected)

	MNI coordinates	Z-score	MNI coordinates	Z-score
	Left hemisphere		Right hemisphere	
**Reading aloud > Fixation**				
Visual cortex	-36 -82 -14	Inf	44 -82 -12	Inf
	-22 -96 -6	Inf	8 -68 -16	7.4
	-20 -66 -22	Inf	22 -96 -2	7.3
	-38 -90 -10	Inf	26 -94 2	7.1
	-28 -92 -12	Inf	28 -88 4	7.1
	-34 -90 -14	Inf	16 -94 -4	6.9
	-38 -88 -14	Inf	18 -98 12	6.8
	-30 -94 2	7.8	14 -98 10	6.8
	-42 -54 -20	7.7	14 -92 2	6.7
	-44 -68 -18	7.5	20 -88 -12	6.6
	-2 -76 -22	7.4	12 -74 12	6.4
Auditory cortex	-60 6 -2	7.7	56 -16 0	Inf
	-62 -20 10	7.4	54 -30 2	7.4
	-52 -36 12	7.2	56 6 -4	7.1
	-62 -14 6	7.1	60 10 -4	7.1
	-60 -30 6	7.0	62 4 -2	7.1
	-50 -22 4	7.0	60 -30 10	7.0
	-60 -10 -2	6.9	60 -8 6	6.9
	-58 -42 12	6.9	66 -16 -2	6.9
	-56 -28 2	6.9	62 -26 4	6.7
	-38 -34 12	6.7	60 -4 -4	6.6
	-46 -34 16	6.6	56 -22 -4	6.4
	-42 -30 12	6.5	44 -30 6	6.3
	-64 -20 -2'	6.4	66 -16 6	6.3
	-66 -26 2	6.4	64 -18 10	6.2
	-52 -34 4	6.0	66 -30 16	6.0
	-44 -38 6	5.9	60 6 -12	5.4
	-58 -36 20	5.9	40 4 -4	5.3
	-48 -42 26	5.8	40 8 -2	5.3
	-30 -34 14	5.3	40 10 2	5.2
			36 4 4	5.2
Motor cortex	-50 -10 34	Inf	52 -6 30	Inf
	-60 -2 14	Inf	58 -4 28	Inf
	-40 -16 40	Inf	60 -4 20	7.7
	-62 -10 20	7.4	54 -8 44	7.2
	-50 -14 42	7.3	64 2 12	6.9
	-58 -10 40	6.9	48 -10 58	6.3
	-46 -8 56	6.8	46 -4 58	6.1
	-48 -12 52	6.7	54 0 46	6.1
	-46 -6 46	6.1		
	-52 4 26	5.6		
Supplementary motor area (SMA)	-4 -4 56	7.4	0 -2 58	7.3
	-4 8 44	6.7	2 -8 64	6.4
	-6 12 40	6.1		
Superior parietal lobule	-28 -54 52	6.4		
	-28 -46 46	5.6		
Cerebellum	-34 -62 -24	Inf	20 -62 -24	Inf
	-46 -62 -28	6.9	42 -60 -26	7.1
	-38 -44 -26	6.8	38 -56 -30	6.9
	-40 -70 -26	6.4	34 -58 -30	6.9
			8 -74 -44	6.7

			8 -70 -38	6.5
Thalamus	-12 -18 -4<br>-12 -18 6	6.7<br>6.6	14 -14 2<br>22 -24 -8<br>8 -20 -10<br>14 -26 -12	7.1<br>6.3<br>5.5<br>5.1
Basal Ganglia	-26 -12 -4<br>-24 -4 4<br>-22 8 2<br>-32 -4 -10<br>-32 -12 12<br>-26 -2 -12	7.8<br>6.5<br>5.7<br>5.5<br>5.4<br>5.2	28 -12 -4<br>24 -6 -4<br>18 4 0<br>24 2 2<br>22 4 12	7.2<br>7.0<br>6.3<br>6.1<br>5.4
**Reading aloud < Fixation**				
Angular gyrus	-44 -78 30<br>-40 -74 36	6.0<br>5.4		
Orbito-frontal cortex	-4 24 -10<br>-4 30 -22	6.4<br>5.5	6 26 -18<br>4 34 -22	6.2<br>5.5
Anterior cingulate cortex	-6 38 -8	5.8	6 38 -4<br>6 50 -4<br>4 44 6	5.8<br>5.8<br>5.5
Anterior middle frontal gyrus	-26 56 0	5.9		
Superior frontal gyrus			26 28 48	6.0

Table S3: Accuracy [%] and RTs [ms] of 15 subjects during reading irregular words and pseudowords.

**Subjects**	**Accuracy [%] Irregular words**	**RT [ms] Irregular words**	**Accuracy [%] Pseudowords**	**RT [ms] Pseudowords**	**RT Difference [ms]: Irregular – Pseudowords**
1	84	800.9	90	1185.6	**-384.75**
2	98	1157.3	100	1529.4	**-372.05**
3	98	931.0	90	1241.6	**-310.63**
4	100	1373.0	100	1613.8	**-240.79**
5	84	1497.9	90	1682.4	**-184.54**
6	100	750.0	90	884.0	**-134.02**
7	76	1786.1	100	1745.5	**40.61**
8	94	1721.5	90	1663.8	**57.75**
9	94	1532.8	95	1448.7	**84.15**
10	100	939.6	100	769.4	**170.21**
11	90	1269.0	95	1051.4	**217.59**
12	98	2132.8	95	1860.4	**272.43**
13	96	1243.0	90	945.7	**297.30**
14	100	1038.4	100	644.9	**393.53**
15	88	1312.3	95	839.7	**472.57**
**mean**	**93.3±7**	**1299±389**	**94.7±4**	**1274±401**	**25±282**

Accuracy was 93.3% for irregular words and 94.7% for pseudowords; mean correct response time was 1299 ms (± 389) for irregular words and 1274 ms (± 401) for pseudowords ($t(14) = 0.35$, $p = 0.73$).
